# Announcing the winners of the 10th anniversary year Short Review competition for early career researchers

**DOI:** 10.14814/phy2.70102

**Published:** 2024-10-31

**Authors:** Josephine C. Adams

**Affiliations:** ^1^ School of Biochemistry University of Bristol Bristol UK

**Keywords:** physiology

## Abstract

Logo for *Physiological Reports'* 10th anniversary year.

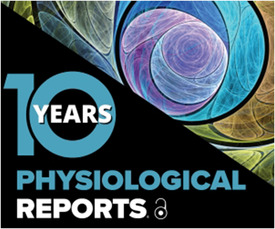

Over the last year, *Physiological Reports* has been celebrating its 10th anniversary year of publication, by expansion of certain journal activities along with inclusion of special content and activities that are unique to the anniversary year (Adams, 2024; Adams et al., 2023). A highlight of the year, which comes to fruition in this issue of *Physiological Reports*, has been to run a Short Review competition targeted to early career researchers. This competition challenged predoctoral and postdoctoral (the latter defined as researchers in the first 5 years after award of the Ph.D.) to write an original, perspective‐type review article related to the general theme of “The Future of Physiological Research.” We asked entrants to tell us about their own ideas on a new or expanding direction of physiological research that they considered will become prominent in 10 years' time, along with their rationales. The article specification was for a maximum of 1500 words and 20 references. A single figure could be included. Relatives or laboratory members of the Editor‐in‐Chief (EiC), Deputy Editor, or Associate Editors were not allowed to enter and the EiC did not take part in the rankings.

The editors were pleased to receive entries from around the world in both the predoctoral and postdoctoral categories. On behalf of all the editors, I thank everyone who entered the competition for their interest and dedication and the scope of the ideas put forward. After initial text‐similarity screening procedures, the anonymized entries were ranked by separate judging panels for each category, according to criteria related to the significance and quality of the scientific rationale, scientific content, presentation and writing style, all based on a scoring matrix. The winners were agreed from these blinded, quantified rankings. I am delighted to introduce here the winners and the topics of their Short Review articles, which are all published in this issue of *Physiological Reports*. For publication, abstracts and a graphical abstract or figure were added to the articles.

In the predoctoral category, the winner is Mr. Kevin John, a graduate student in the laboratory of Prof. Julien D Périard at the Research Institute for Sport and Exercise, University of Canberra, Australia. Mr. John's topic is “Hot pants: The emerging field of exercise mimetics, from hospital beds to the international space station” (John, 2024).

In the postdoctoral category, the winner is Dr. Josh Thorley, School of Sport, Exercise, and Health Sciences, University of Loughborough, UK. His article is entitled “Unravelling the redox code to improve physiological research in human health and disease” (Thorley, 2024). Given the overall strength of the entries in this category, the editors chose to make an additional “Highly commended” award to the second‐ranked article in the post‐doctoral category. This award goes to Dr. Lorcan Daly, Dept. of Sport and Health Sciences, Technological University of the Shannon, Ireland, for his article “The Future of Physiological Research: A Greater Understanding of Female Master Athletes and Ageing?” (Daly, 2024).

For the editors, it was a very enjoyable and interesting experience to read all the entries. It is notable that, fortuitously, the winning articles show diverse perspectives that encompass both basic and translational areas of physiology and also relate to the breadth of research covered by *Physiological Reports*. I encourage all readers to take the time to consider these short reviews, which speak not only to the future of physiological research but also to the curiosity, talent and energy of researchers who are currently building their careers and who will carry the field forward in the future.

## FUNDING INFORMATION

No funding was associated with the preparation of this Editorial.

## ETHICS STATEMENT

The author is the Editor‐in‐Chief of *Physiological Reports* and was blinded from reviewing or making decisions for this manuscript. An alternate editor oversaw the manuscript process for this article.

## Data Availability

The data can be found within this article.
